# A topical nociceutical formulation ameliorates chemotherapy-induced peripheral neuropathy: a pilot randomized clinical study

**DOI:** 10.1007/s12094-025-04062-1

**Published:** 2025-10-01

**Authors:** Sonia Servitja, Maria Castro-Henriques, Iñaki Álvarez-Busto, Carlota Díez-Franco, Alba Medina-Castillo, Maria Asunción Algarra-García, Elena López-Miranda, Margaret Lario-Martínez, Maria Isabel Luengo-Alcázar, Miguel Borregón, Ana Davó, Anna Gasull-Delgado, Sara Roque-García, Ana Gonzaga-López, Jesús Manuel Poveda-Ferriols, Severine Pascal, Ana María Mitroi-Marinescu, Marta García-Escolano, Asia Fernández-Carvajal, Clotilde Ferrándiz-Huertas, Antonio Ferrer-Montiel

**Affiliations:** 1 Servicio de Oncología Médica, Hospital del Mar, 08003 Barcelona, Spain; 2 Servicio de Oncología Médica, Hospital Universitario Miguel Servet, 50009 Saragossa, Spain; 3 Aragon Health Research Institute (IIS Aragón), Saragossa, Spain; 4 Servicio de Oncología Médica, Hospital Marina Baixa, 03570 Villajoyosa, Alicante Spain; 5 Servicio de Oncología Médica, Hospital Universitario Ramon y Cajal, 28034 Madrid, Spain; 6 Servicio de Oncología Médica, Hospital General Universitario Santa Lucía, 30202 Cartagena, Murcia Spain; 7 Hospital General Universitario de Elda, 03600 Elda, Alicante, Spain; 8 Servicio de Oncología Médica, Hospital General Universitario de Elche, 03202 Elche , Alicante Spain; 9 Servicio de Oncología Médica, Hospital Universitari Sagrat Cor, 08029 Barcelona, Spain; 10 CHU St-Pierre-UMC St-Pieter, 1000 Brussels, Belgium; 11 Scientific Park, Prospera Biotech, University Miguel Hernández, 03202 Elche, SL Spain; 12 Instituto de Investigación, Desarrollo E Innovación en Biotecnología Sanitaria de Elche (IDiBE), Universidad Miguel Hernández, 03202 Elche, Spain

**Keywords:** CIPN, Nociception, Capsaicin, Epidermal endings, Neuropathy

## Abstract

**Background:**

Up to 80% of patients undergoing taxanes or platinum-based chemotherapy (CT) develop a peripheral polyneuropathy (CIPN), that affects treatment compliance and quality of life (QoL). CIPN is characterized by a remarkable sensitization of peripheral nociceptive endings. We performed a proof-of-concept, double-blind, randomized, two-arms, multicenter clinical study to evaluate if protecting epidermal nociceptive endings with a topical nociceutical formulation prevented CIPN and augmented QoL during CT.

**Material and methods:**

Participants started a daily topical application of the assigned formulation in hands (moisturizing or nociceutical). Upon appearance of neuropathic symptoms in hands or feet, they applied the creams twice daily. Diagnosis and follow-up of CIPN was performed using the CTC AE v5.0 criteria.

**Results:**

A cohort of 142 patients treated with taxanes and/or platinum agents were randomly distributed into the arms. Withdrawals were similar in both arms. A lower CIPN incidence in hands was observed in the nociceutical arm (32% vs 13%, *p* = 0.03), while a similar number of participants developed CIPN in feet (73% vs 67%, *p* = 0.1). Interestingly, the nociceutical formulation increased the number of CT cycles CIPN free (6 vs 8 cycle, *p* = 0.009). The Leonard Scale Questionnaire revealed that 60% of patients using the moisturizing cream reported frequently bothersome neuropathic symptoms, compared with only 39% in the nociceutical group (*p* = 0.0017).

**Conclusion:**

Protection of nociceptive epidermal terminals with a topical nociceutical formulation reduced the incidence of CIPN in hands and increased the QoL of patients. These findings provide a solid ground for a confirmatory clinical study.

## Introduction

Chemotherapy‐induced peripheral neuropathy (CIPN) is a prevalent and debilitating adverse effect observed in up to 80% of cancer patients receiving taxanes or platinum agents [1]. Clinically, CIPN manifests as a range of distressing sensory disturbances and motor deficits. These symptoms significantly impair quality of life (QoL) and may require modifications in chemotherapeutic regimens, ranging from dose reductions to treatment cessation [1]. Typically, CIPN becomes evident around the fourth to sixth cycle of chemotherapy and intensifies with cumulative dosing [2]. Moreover, in approximately 30–40% of patients, these neuropathic symptoms may persist for 6 months or longer following treatment cessation [3]. Despite the profound impact of CIPN on both patient well‐being and therapeutic outcomes, there remains a paucity of effective prophylactic or ameliorative strategies. While low‐grade (I/II) neuropathy is often managed with sensitive skin creams, high‐grade (III/IV) manifestations are treated palliatively with agents such as duloxetine, gabapentin or Qutenza® patches containing 8% capsaicin [4]. Preventative measures, including the use of cold gloves to limit chemotherapy exposure to distal epidermal regions, along with physical exercise and physiotherapy, have been explored; however, the supporting evidence remains variable.

Complementarily, studies have evaluated the benefits of exercise and resistance training [5], cryotherapy and compression therapy [6], wireless cutaneous nerve stimulation [7], acupuncture [8], intravenous monosialotetrahexosylganglioside (GM‐1) [9], omega‐3 fatty acids and essential oils [10, 11], and even oral lafutidine [12]. Notably, the American Society of Clinical Oncology (ASCO) guidelines endorse only those interventions with robust clinical support, such as duloxetine, gabapentin, and Qutenza® patches [13]. Thus, an unmet clinical need persists for approaches that can prevent or attenuate CIPN, thereby preserving both supportive care and treatment compliance [14].

Pre‐clinical investigations have implicated the excitation of distal epidermal sensory terminals by chemotherapy as a primary mechanism underlying CIPN [15]. A direct action on sensory nerve endings, along with alteration of cutaneous and immune cells, results in the sensitization of nociceptor endings. In addition, Schwann cell toxicity can precipitate deficits in myelination and foster the chronification of neuropathic symptoms [16]. Such cellular alterations enhance the excitability of nociceptors, leading to spontaneous electrical discharges that characterize CIPN [17]. Among the molecular mediators, thermosensitive channels such as TRPV1, TRPM8, and TRPA1, as well as voltage‐gated sodium channels, have emerged as potential targets for protecting epidermal nociceptive terminals [17–19]. In vitro studies demonstrate that exposure of sensory neurons to paclitaxel or oxaliplatin for 24–48 h significantly increases both spontaneous and evoked electrical activity, predominantly through potentiation of TRPV1 channel activity [17]. This evidence underpins the rationale for exploring topical strategies aimed at safeguarding nociceptive epidermal terminals to prevent the onset and ameliorate the severity of CIPN.

Nociceuticals represent a novel class of topical, moisturizing formulations that incorporate active compounds designed to protect nociceptive epidermal endings. By exerting a biological activity through a multitarget action, these formulations may reduce the underlying neuronal excitability responsible for CIPN. In this context, we have developed a family of soft vanilloid-based compounds that interact with thermosensory channels and receptors present in the epidermal nociceptive endings [20, 21]. These compounds are engineered with an esterase‐sensitive ester group, ensuring hydrolysis within the dermis and preventing systemic distribution. Pre‐clinical evaluation have shown that these soft vanilloids can significantly reduce thermal hyperalgesia and itch [21]. Collectively, these observations suggest that soft vanilloids may help in the topical management of CIPN.

To test this hypothesis in a clinical setting, we designed a pilot, double‐blind, randomized, multicenter clinical study to assess the protective efficacy of a nociceutical formulation. This formulation combines a non‐pungent soft vanilloid (Calmapsin®) with lipids and tocopherol, to protect nociceptive epidermal terminals and their environment. As a comparator, a base moisturizing formulation was used. We report that the prophylactic application of the nociceutical formulation in the hands reduced the incidence and the severity of CIPN, improving patient QoL. Moreover, it delayed CIPN onset during CT, consistent with a therapeutic benefit of protecting epidermal nociceptive endings. These promising results support a larger, treatment-stratified, confirmatory clinical study.

## Material and methods

### Patients and study design

This proof‐of‐concept study was conducted as a multicenter, double‐blind, randomized, placebo‐controlled trial across nine hospitals, under the auspices of Clinical Trials registration NCT06733545 (submitted: November 26, 2024). The study protocol (reference 20-PB-02) received approval from the Ethics Committee of Hospital General Universitario de Elche (Code: PI 62/2020) and validated by the Ethics Committees of all participating hospitals, and informed consent was obtained from all patients prior to enrollment. The study was performed in accordance to the Declaration of Helsinki.

Eligible patients were required to be adults (≥ 18 years) diagnosed with stage I–III primary cancer and scheduled to commence chemotherapy regimens incorporating taxanes and/or platinum agents. Patients were carefully screened against the inclusion and exclusion criteria (Table 1) by medical oncologists, who also assumed responsibility for obtaining informed consent, ensuring eligibility, collecting data in standardized booklets, and monitoring for the onset and progression of CIPN.

**Table 1:** Inclusion–exclusion criteria used to recruit the patient cohort

Inclusion criteria	Exclusion criteria
Age ≥ 18 year	Using antidepressants or immunosuppressants
Capacity or complete questionnaires	Suffering peripheral neuropathies
Sign informed consent	Suffering neuromuscular disease
Capacity to self-apply the formulations	Suffering cardiac disease
Diagnosed with stage I–III primary cancer	Being involved in a clinical trial
Starting CT or having received 1 CT cycle at most	Exhibiting brain metastasis
CT with taxanes or platinum agents or both	Using topical medications
ECOG performance status: 0–2	Exhibiting sensitivity to capsaicin
Life expectancy ≥ 6 months	Having a history of neuropathies

### Randomization procedure

Randomization was performed using a computer‐generated random number sequence with a block size of ten, and stratification was applied according to the study center to ensure balanced allocation. Following randomization, each patient was assigned a unique code and dispensed one of two identically appearing formulations to ensure blindness: the base moisturizing cream (labeled as PB‐011) or the nociceutical formulation (labeled as PB‐012).

### Procedures and assessments

Patients were instructed to apply the assigned cream topically to their hands once daily at the initiation of chemotherapy. Upon the emergence of any sensory discomfort—such as tingling, itching, or altered thermal sensations—in the hands and/or feet, patients were advised to increase application frequency to twice daily on both areas. The onset and severity of CIPN were diagnosed and monitored by the study investigators using the National Cancer Institute’s Common Terminology Criteria for Adverse Events (CTCAE) version 5.0. In addition, patients were required to complete the Leonard Scale Questionnaire every 3 weeks, up to 1 month after the conclusion of chemotherapy, to quantify the impact of neuropathic symptoms on daily activities. Symptom severity was categorized as “hardly at all bothered” (score 0–1), “moderately bothered” (score 2) or “extremely bothered” (score ≥ 3) [22].

### End points

The primary endpoint of the study was the percentage of patients who remained free of CIPN throughout the chemotherapy course. Secondary endpoints included the proportion of chemotherapy cycles during which patients remained free of neuropathic symptoms, the degree of symptom severity reduction and adherence to the prescribed chemotherapy regimen, as evidenced by any dose reductions or treatment cessations necessitated by CIPN.

### Adverse effects

Adverse events (AEs) were diligently monitored by the investigators at each follow‐up visit and were recorded in accordance with CTCAE v5.0 criteria. The only formulation‐related AE observed was mild pruritus (grade 1), which was reported in four patients (two in each arm). All other adverse events were attributable to the chemotherapeutic agents and were managed as per standard clinical protocols.

### Topical formulations

The topical formulations were prepared and provided by the study sponsor in identical white tubes to preserve blinding. The base moisturizing cream comprised a blend of capric triglyceride, glyceryl behenate, glyceryl stearate, hydrogenated castor oil, diethylene glycol monoethyl ether, and tocopherol. The nociceutical formulation (Oncapsisens®) contained these same constituents with the addition of hydroxymethoxyiodobenzyl glycolamide perlargonate, which was specifically incorporated to reinforce the modulatory effect on thermosensory nociceptive terminals.

### Statistical analyses

The sample size was calculated using the Wilcoxon–Mann–Whitney test (two‐tailed, effect size *d* = 0.60) to achieve a statistical power of 0.95 at an alpha level of 0.05. An initial estimate of 120 participants (60 per arm) was adjusted to 140 patients to account for an anticipated dropout rate of 20%. Differences between groups for continuous variables (e.g., percentage of chemotherapy cycles free of CIPN) were analyzed using the non‐parametric Mann–Whitney rank sum test. Categorical data, including responses from the Leonard Scale Questionnaire and the incidence of CIPN in specific body regions, were compared using Fisher’s exact test or the Chi‐square test, as appropriate. All statistical analyses were performed using GraphPad Prism 10.

## Results

**Fig. 1:**
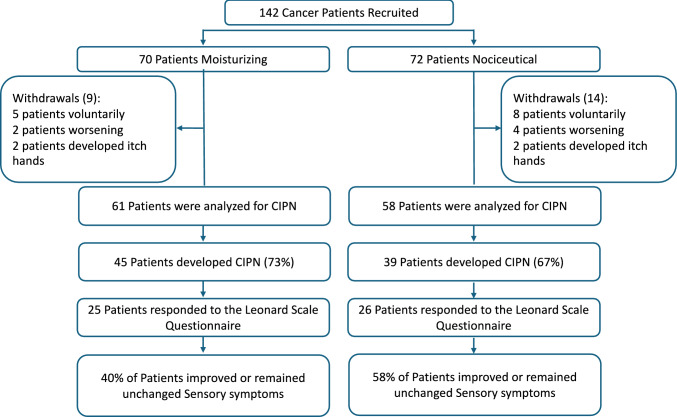
Consort diagram

A total of 142 cancer patients (127 women and 15 men, mean age 56 ± 14 years) were enrolled in the study (Fig. 1, Tables 1 and 2). Most patients were diagnosed with breast cancer, with smaller proportions presenting gastrointestinal or gynecological tumors. Approximately 72–74% of the patients received taxane‐based chemotherapy, predominantly for breast cancer, while a minority received platinum agents or a combination of taxanes and platinum compounds (Table 2).

**Table 2:** Cohort of patients

	Moisturizing	Nociceutical
Demographic profile		
Number of patients	70	72
Females	63	65
Males	7	7
Age (years, mean ± SD)	55 ± 11	57 ± 14
Cancer type (%)		
Breast	84	76.3
Colon	4.2	6.9
Rectal	2.9	4.2
Gastric	4.4	2.8
Endometrial	1.5	4.2
Ovarian	0	2.8
Pancreatic	1.5	1.4
Nasopharyngeal	0	1.4
Testicular	1.5	0
Comorbidities (%)		
Metabolic	30.5	32.2
Endocrine	11	13.1
Diabetes	9.8	8.2
Lung	8.5	8.2
Neurologic	8.5	6.6
Digestive	6.0	3.3
Cardiovascular	3.7	1.6
Bone	3.7	4.9
Kidney	2.4	1.6
None	15.8	21.4
Chemotherapy (%)		
Taxanes-based	72.1	74.1
Platinum agents-based	14.8	8.6
Taxanes and platinum agents	13.1	17.3
CT duration (no. cycles, mean ± SD)	10.2 ± 3	10 ± 3
CT dose reduction due to CIPN	2 (taxanes)	2 (taxanes/platinum agents)
CT cessation due to CIPN	3 (taxanes)	2 (taxanes)
Grade I CIPN (no. Patients)	30	27
Grade II CIPN (no. Patients)	12	10

Following randomization, 70 patients were assigned to receive the base moisturizing formulation and 72 to receive the nociceutical formulation. During the study, withdrawals occurred in both arms—9 patients in the moisturizing group and 14 in the nociceutical group. Withdrawals were attributable to various reasons including tumor progression (two and three patients per group, respectively), voluntary discontinuation (five in the moisturizing arm and nine in the nociceutical arm), and the onset of pruritus upon cream application (two patients in each arm). Consequently, 61 patients in the moisturizing group and 58 in the nociceutical group were evaluable for the primary endpoint.

**Fig. 2:**
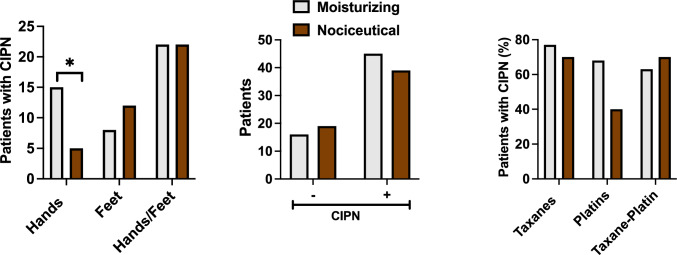
The use of the nociceutical formulation prevents CIPN in hands.** a** Patients that developed CIPN symptoms in both arms. **b** Patients that received taxanes and platinum agents that developed the neuropathy. **c** Percentage of patients that developed the neuropathy in hands, feet, and hand–feet. Patients started to use the formulation once daily in hands at the beginning of the CT. Upon CIPN manifestation in feet or hands, patients applied the formulation twice daily. Data represent the percentage of patients that developed CIPN at any CT cycle of the 12 cycles considered in this study. For each arm, patients were normalized by the total cohort analyzed for CIPN, excluding patients that withdrawn the study. Statistical analysis was performed with the Fisher’s exact test, *p = 0.0307, 95% CI from 0.3438 to 0.9514, OR 0.2857. Only significant differences are indicated

Patients of both arms started using the formulations once a day in hands at the beginning of the prescribed CT. Application of the creams was monitored, on average, for 10 ± 3 CT cycles. Both formulations were safe only exhibiting as AEs the manifestation of itch (grade 1, CTC AE v5.0) in hands upon application in four patients (two in each arm). This reaction may be due to ethoxydiglycol (Transcutol®) present in the formulations. All other AEs were related with the CT and recorded by study investigators using the CTC AE v5.0. Analysis of the CIPN incidence in the hands showed a statistically significant reduction in the nociceutical arm (32% vs. 13%, *p* = 0.0307, 95%CI from 0.3438 to 0.9514, OR = 0.2857, two-tailed Fisher´s exact test) (Fig. 2a). The overall incidence considering the CIPN in the feet revealed a reduced tendency to develop neuropathic symptoms during chemotherapy (73% vs. 67%, p = 0.1, Fig. 2b). In both arms, a similar proportion of patients required CT dose reductions or cessation due to CIPN (Table 2). Patients receiving taxanes or taxanes and platinum agents exhibited a similar CIPN incidence in both groups (70%, Fig. 2c). Notably, 68% patients receiving platinum agents (FOLFOX, CAPOX or XELOX) exhibited CIPN in the moisturizing arm and 40% in the nociceutical arm, although the limited number of patients getting platinum agents prevents a proper statistical analysis.

**Fig. 3:**
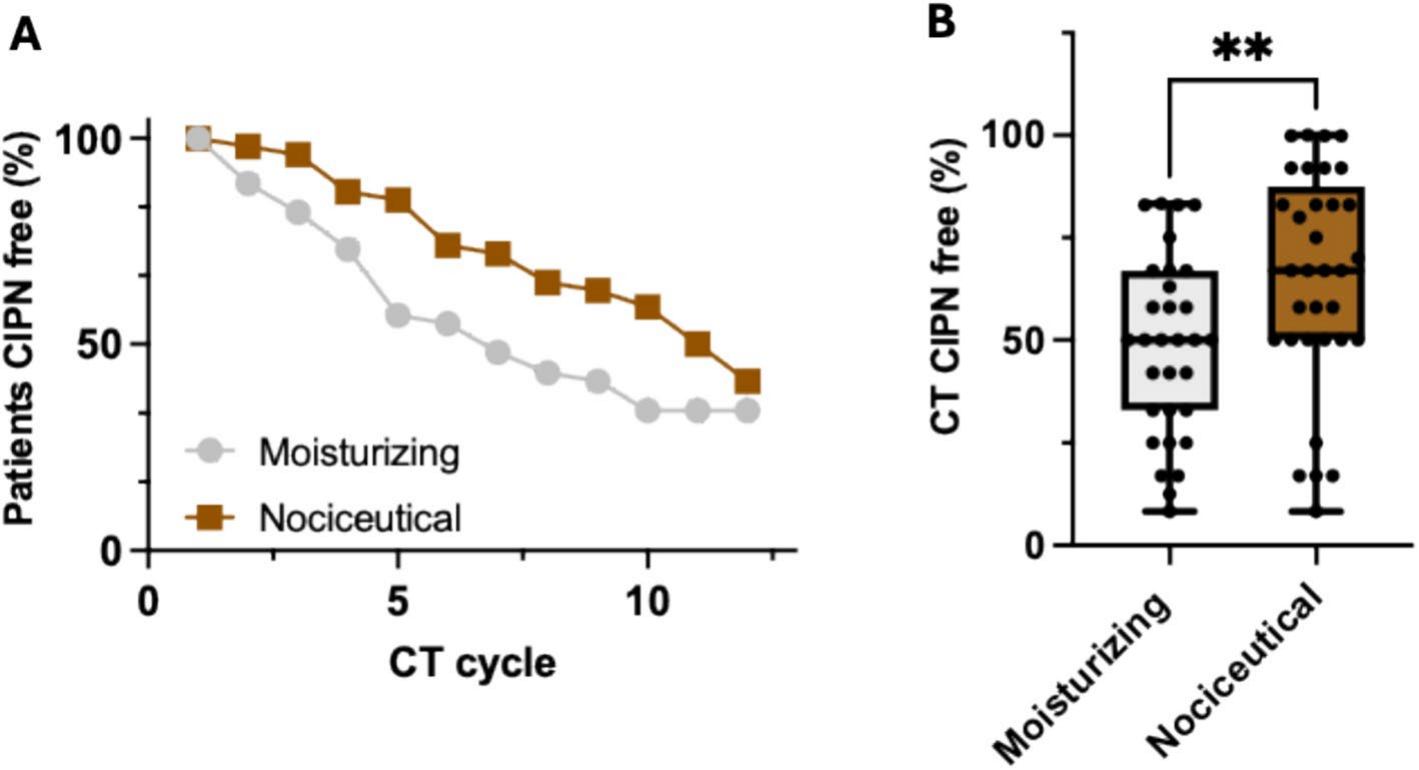
The nociceutical formulation delays the onset of CIPN as compared with the moisturizing formulation.** a** Percentage of patients CIPN free throughout the 12 chemotherapy cycles. **b** Percentage of the CT that patients developing CIPN are free of sensory symptoms. Data are mean ± SD, with *n* = 31 and 33 patients for the moisturizing and nociceutical arms, respectively (Fig. 1). Statistical analysis was performed using the non-parametric Mann–Whitney rank sum test. **p = 0.0091 (sum or ranks 651 and 1002; *U* = 245; 95% CI from 33 to 63 and 50 to 83)

In addition to the incidence data, we noted that the onset of neuropathic symptoms, assessed by the percentage of CT cycles free of CIPN, was significantly delayed in the nociceutical arm (Fig. 3a). While patients in the moisturizing arm exhibited 49 ± 22% (mean ± SD) of CT cycles free of CIPN, patients of the nociceutical arm remained 65 ± 27% (mean ± SD) of the CT cycles without neuropathic symptoms (*p* = 0.0096, two-tailed Mann–Whitney rank sum test) (Fig. 3b). This finding suggests that prophylactic use of the nociceutical formulation not only reduces the incidence of CIPN in the hands, but also postpones its clinical onset on a group of patients developing the neuropathy.

**Fig. 4:**
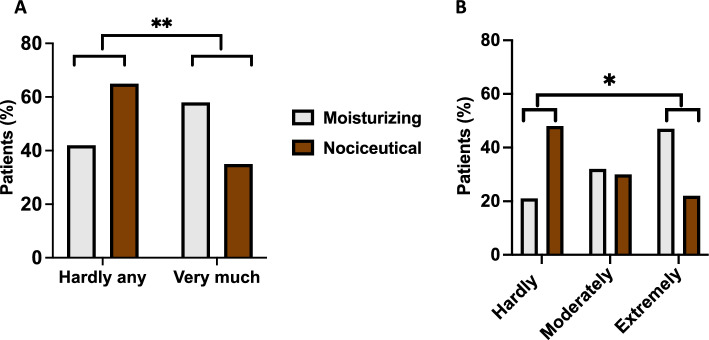
The use of the nociceutical formulation reduces the severity of the CIPN sensory symptoms. **a** Percentage of patients that indicate they experienced hardly any symptom or very much disturbing sensory symptoms in hands and/or feet. Statistical comparison was performed using the Fisher´s exact test: **p = 0.0017, 95% CI from 0.4709 to 0.8321, OR = 0.39. *N* = 23 and 25 for moisturizing and nociceutical formulations, respectively **b** Percentage of patients indicating whether the sensory symptoms hardly, moderately, or extremely bothered them performing daily activities. The severity of the sensory symptoms was divided as hardly at all bothered (0–1), moderately bothered (2), and extremely bothered (≥ 3) as indicated by Leonard et al. [22]. Statistical comparison was performed using the Contingency test Chi-square for trend, *p = 0.0418. Only significant differences are indicated

The impact of sensory symptoms on daily activities was further evaluated using the Leonard Scale Questionnaire [22]. Among the respondents, 60% of patients using the moisturizing cream reported frequently bothersome neuropathic symptoms, compared with only 39% in the nociceutical group (*p* = 0.0017, 95%CI from 0.4709 to 0.8321, OR = 0.39, two-tailed Fisher´s exact test) (Fig. 4a). When categorizing the severity of the symptoms, a significant shift was observed in the nociceutical arm, with a higher proportion of patients reporting that the sensory symptoms “hardly at all” interfered with their daily activities, as opposed to those experiencing moderate or extreme disruption (*p* = 0.0418, Chi‐square test for trend) (Fig. 4b).

Collectively, these results indicate that the nociceutical formulation conferred a benefit by reducing the incidence in the hands, delaying the onset of neuropathic symptoms, and reducing their severity, particularly in the hands, thereby enhancing patient QoL.

## Discussion

CIPN remains a major challenge in oncological practice, as its occurrence can markedly diminish patient QoL and compromise the continuity and efficacy of chemotherapy regimens. The neuropathy is principally driven by the direct excitation of peripheral nociceptive terminals by chemotherapeutic agents, a process that may be further amplified by their microenvironment, i.e., molecules released by cutaneous, immune, and Schwann cells [15–17]. In recent years, there has been growing interest in targeting thermosensory ion channels as a means of modulating CT-induced nociceptive excitability [17–19, 23, 24]. Our multicenter, double‐blind, randomized pilot trial provides evidence supporting the clinical value of a prophylactic topical strategy, modulating these channels to protect peripheral nociceptive endings during CT. Patients who received the nociceutical formulation in hands at the beginning of CT experienced a significant reduction of palmar CIPN in comparison to those treated with the base moisturizing cream. Moreover, patients in the nociceutical group remained free of neuropathic symptoms for a greater proportion of their CT cycles. In addition, patients using the nociceutical cream reported that neuropathic symptoms were less disruptive to their daily activities. This reduction in symptom severity is of paramount importance, as even low‐grade neuropathy can significantly impair functional status and overall well‐being [4]. The observed improvement in patient‐reported outcomes highlights the potential of topical nociceutical formulations to serve as a valuable companion to existing supportive care measures.

When compared with established pharmacological interventions for CIPN, such as duloxetine, gabapentin, and Qutenza® patches, our topical approach offers several potential advantages. Current systemic treatments are typically reserved for high‐grade neuropathy and are often accompanied by a range of side‐effects that limit their clinical utility [4]. In contrast, a topical formulation that acts directly on nociceptive epidermal endings offers a more targeted and safer alternative. The mechanistic basis for this approach is supported by pre‐clinical data demonstrating that vanilloid-based soft antagonists reduce nociceptive sensitization by modulating thermosensory epidermal channels [20, 21]. In addition, the incorporation of lipids and tocopherol further enhance a protective effect by synergistically modulating membrane receptor activity and exerting antioxidant actions [25–27].

Despite these promising findings, several limitations of the study must be acknowledged. The study cohort was predominantly composed of women with breast cancer receiving taxane‐based chemotherapy, thereby limiting the generalizability of the results to other cancer types and chemotherapeutic regimens. Moreover, the relatively small sample size, coupled with a limited number of patients completing the Leonard Scale Questionnaire, necessitates cautious interpretation of the data. Future studies should endeavor to include a more diverse patient population and utilize additional QoL instruments, such as the EORTC QLQ‐CIPN20, to capture a broader spectrum of physical, emotional, and cognitive functions [28, 29]. Because of the safety profile of the formulation, it is also conceivable that increasing the frequency of topical applications from the outset of chemotherapy may yield further protective benefits, an aspect that warrants further investigation in subsequent trials.

## Conclusion

We report that a prophylactic topical formulation could reduce the incidence and severity of epidermal CIPN, supporting the tenet that direct protection of peripheral nociceptive endings may help managing CIPN [24, 30, 31]. While a confirmatory, larger clinical trial is required to validate these findings and to establish the optimal dosing regimen, our results indicate that a topical intervention could complement existing pharmacological therapies and offer a safe, local approach to ameliorate the burdensome of epidermal CIPN and increase the QoL of cancer patients during and post chemotherapy.

## Data Availability

Data will be shared upon request to corresponding authors.
