# Correction: A topical nociceutical formulation ameliorates chemotherapy‑induced peripheral neuropathy: a pilot randomized clinical study

**DOI:** 10.1007/s12094-025-04116-4

**Published:** 2025-11-28

**Authors:** Sonia Servitja, Maria Castro-Henriques, Iñaki Álvarez-Busto, Carlota Díez-Franco, Alba Medina-Castillo, Maria Asunción Algarra-García, Elena López-Miranda, Margaret Lario-Martínez, Maria Isabel Luengo-Alcázar, Miguel Borregón, Ana Davó, Anna Gasull-Delgado, Sara Roque-García, Ana Gonzaga-López, Jesús Manuel Poveda-Ferriols, Severine Pascal, Ana María Mitroi-Marinescu, Marta García-Escolano, Asia Fernández-Carvajal, Clotilde Ferrándiz-Huertas, Antonio Ferrer-Montiel

**Affiliations:** 1 Servicio de Oncología Médica, Hospital del Mar, 08003 Barcelona, Spain; 2 Servicio de Oncología Médica, Hospital Universitario Miguel Servet, 50009 Saragossa, Spain; 3 Aragon Health Research Institute (IIS Aragón), Saragossa, Spain; 4 Servicio de Oncología Médica, Hospital Marina Baixa, 03570 Villajoyosa, Alicante Spain; 5 Servicio de Oncología Médica, Hospital Universitario Ramon y Cajal, 28034 Madrid, Spain; 6 Servicio de Oncología Médica, Hospital General Universitario Santa Lucía, 30202 Cartagena, Murcia Spain; 7 Hospital General Universitario de Elda, 03600 Elda, Alicante, Spain; 8 Servicio de Oncología Médica, Hospital General Universitario de Elche, 03202 Elche , Alicante Spain; 9 Servicio de Oncología Médica, Hospital Universitari Sagrat Cor, 08029 Barcelona, Spain; 10 CHU St-Pierre-UMC St-Pieter, 1000 Brussels, Belgium; 11 Scientific Park, Prospera Biotech, University Miguel Hernández, 03202 Elche, SL Spain; 12 Instituto de Investigación, Desarrollo E Innovación en Biotecnología Sanitaria de Elche (IDiBE), Universidad Miguel Hernández, 03202 Elche, Spain

**Correction: Clinical and Translational Oncology (2025)** 10.1007/s12094-025-04062-1

In this article the Author contributions section omitted the contributions of author Clotilde Ferrándiz-Huertas, specifically in regard to the collection and assembly of data. The contributions statement was incorrectly given as “Conception and design: SS, AFM. Administrative support: all authors. Collection and assembly of data: SS, MCH, IAB, CDF, AMC, MAAG, ELM, MLM, MILA, MB, AD, AGD, SRG, AGL, JMPF, SP, AMMM. Data analysis and interpretation: SS, CFH, AFM. Manuscript writing: SS, AFM. Revision and approval: all authors. Accountability: all authors” but should have been as follows:

**Author contributions**: Conception and design: SS, AFM. Administrative support: all authors. Collection and assembly of data: SS, MCH, IAB, CDF, AMC, MAAG, ELM, MLM, MILA, MB, AD, AGD, SRG, AGL, JMPF, SP, AMMM, CFH. Data analysis and interpretation: SS, CFH, AFM. Manuscript writing: SS, AFM. Revision and approval: all authors. Accountability: all authors.

The original article has been corrected.

